# Study of the Lipid Profile of ATCC and Clinical Strains of *Staphylococcus aureus* in Relation to Their Antibiotic Resistance

**DOI:** 10.3390/molecules24071276

**Published:** 2019-04-02

**Authors:** Carlo Bisignano, Giovanna Ginestra, Antonella Smeriglio, Erminia La Camera, Giuseppe Crisafi, Flavio A. Franchina, Peter Q. Tranchida, Angela Alibrandi, Domenico Trombetta, Luigi Mondello, Giuseppina Mandalari

**Affiliations:** 1 Department of Biomedical, Dental, Morphological and Functional Images Sciences, University of Messina, Via C. Valeria, 98125 Messina, Italy; cbisignano@unime.it; 2 Department of Chemical, Biological, Pharmaceutical and Environmental Sciences, University of Messina, I-98168 Messina, Italy; gginestra@unime.it (G.G.); asmeriglio@unime.it (A.S.); elacamera@unime.it (E.L.C.); gcrisafi@unime.it (G.C.); ptranchida@unime.it (P.Q.T.); dtrombetta@unime.it (D.T.); lmondello@unime.it (L.M.); 3 Chromaleont c/o Department of Chemical, Biological, Pharmaceutical and Environmental Sciences, University of Messina, I-98168 Messina, Italy; ffranchina@unime.it; 4 School of Engineering at Dartmouth College, 14 Engineering Drive, Hanover, NH 03755, USA; 5 University of Liège, Molecular System Organic & Biological Analytical Chemistry, 11 Allée du Six Août, 4000 Liège, Belgium; 6 Department of Economics, Unit of Statistical and Mathematical Sciences, University of Messina, 98125 Messina, Italy; aalibrandi@unime.it

**Keywords:** *Staphylococcus aureus*, lipids, antibiotic resistance, hydrophobicity, ATCC strains, clinical strains

## Abstract

A number of reports have indicated a relationship between bacterial resistance to antibiotics and their lipid composition. In the present study, we characterized the lipid profiles of American Type Culture Collection (ATCC) and clinical strains of *Staphylococcus aureus* and its correlation with antibiotic resistance and hydrophobicity. The following strains were used: *S. aureus* ATCC 6538P, *S. aureus* ATCC 43300 (MRSA), seven clinical strains from the pharynges, two strains from duodenal ulcers, four strains from hip prostheses, and one strain from the conjunctiva. Lipid-related differentiation was observed across the *S. aureus* strains: the higher abundance of *anteiso*-pentadecanoic acid (*anteiso*-C_15:0_) and *anteiso*-heptadecanoic acid (*anteiso*-C_17:0_), followed by *iso*-pentadecanoic acid (*iso*-C_15:0_), suggested that these were common lipids. *Iso*-tridecanoic acid (*iso*-C_13:0_) and *anteiso*-tridecanoic acid (*anteiso*-C_13:0_), *iso*-hexadecanoic acid (*iso*-C_16:0_) and *anteiso*-hexadecanoic acid (*anteiso*-C_16:0_), and all forms of octadecanoic acid (C_18:0_) were usually detected in low abundance. Strains isolated from pharynges showed the highest ratio of branched/straight chains. A distinction in two clusters based on the amount and type of bacterial lipids identified was obtained, which correlated to the antibiotic resistance, the strains origin, and the cell-surface hydrophobicity. We report a potential correlation between the lipid profile of *S. aureus* strains, site of infection, antibiotic resistance, and cell-surface hydrophobicity. These results, which still need further insights, could be a first step to identifying antibiotic resistance in response to environmental adaptation.

## 1. Introduction

Amongst the significant Gram-positive human pathogens, *Staphyloccus aureus* (*S. aureus*) and methicillin-resistant *S. aureus* (MRSA) play an important role, being responsible for a variety of infections, such as skin, respiratory, and bone joint infection, as well as endocarditis, bacteremia, and toxic shock syndrome [1]. The ability of *S. aureus* to produce a biofilm makes this strain responsible for infections of implanted medical devices, wounds associated infections, cystic fibrosis, and otitis media [2,3,4]. Due to the increased spread of multi-drug resistance, growing interest has been focused on the identification of novel antimicrobial agents against *S. aureus* and MRSA: a number of natural compounds and antimicrobial peptides have been identified [5,6,7]. Over the last few decades, understanding the mechanisms involved in the adaptation of microbial cells to environmental conditions has gained much interest [8]. Exposure to specific antimicrobials at sub-lethal concentrations could result in acquired resistance [9,10]. 

The relation between bacterial resistance to different antibiotics and lipid composition of these organisms has been reported: Yehia et al. [11] have demonstrated that antibiotic treatment determined a genetic alteration of the lipid biosynthesis in *Pseudomonas aeruginosa*, with an effect on exopolysaccharides production. The lipid profiles of three closely related strains of *Escherichia coli* were monitored using high resolution mass spectrometry: results showed a shift in the lipid distribution that correlated to the antibiotic exposure [12]. Furthermore, changes in the lipopolysaccharide profile of clinical isolates of *Porphyromonas gingivalis* correlated with changes in colony morphology and resistance to polymyxin B in both healthy and periodontitis patients: this trend was associated with differences in bacterial virulence and progression of periodontitis [13]. The characterization of cell membrane parameters of clinical isolates of *S. aureus* with different susceptibility to the alpha-melanocyte stimulating hormone demonstrated the presence of a rigid membrane. Higher amounts of the cationic phospholipid were detected in the strains resistant to the peptide [7]. 

We have previously characterized the lipid profiles of five different strains of Gram-positive and Gram-negative bacteria using a rapid sample preparation method and fast comprehensive two-dimensional gas chromatography in combination with mass spectrometry (GC×GC-MS) [14]. In the present paper, we report the characterization of the lipid profiles of ATCC and clinical strains of *S. aureus* and their correlation with antibiotic resistance and hydrophobicity: the origin of the clinical strains was also be evaluated in relation to drug resistance.

## 2. Results

### 2.1. Bacterial Lipid Profiles

The identification of BAMEs and their relative abundance across the tested strains is reported in Table 1. Lipid-related differentiation across the various *S. aureus* strains was observed: the higher abundance of *anteiso*-pentadecanoic acid (*anteiso*-C_15:0_) and *anteiso*-heptadecanoic acid (*anteiso*-C_17:0_), followed by *iso*-pentadecanoic acid (*iso*-C_15:0_), suggested that these lipids were common and could be detected in all strains. However, other lipids, including *iso*-tridecanoic acid (*iso*-C_13:0_) and *anteiso*-tridecanoic acid (*anteiso*-C_13:0_), *iso*-hexadecanoic acid (*iso*-C_16:0_) and *anteiso*-hexadecanoic acid (*anteiso*-C_16:0_), and all forms of octadecanoic acid (C_18:0_), were usually detected in low abundance and did not appear in all strains. Strangely, no *iso*-C_15:0_ branched chain fatty acid was detected in strain 98 isolated from conjunctiva. The lipid composition of strain 530 was unique, with the lowest concentration of the most abundant fatty acid present, *anteiso*-C_15:0_, and the highest concentration of the polyunsaturated C_18:3_ (n-3). Table 2 reports some features of the lipid profiles detected in the bacterial strains. Interestingly, strains isolated from pharynges showed the highest ratio of branched/straight chain, with the exception of strain 530, followed by strain 8 and the MRSA ATCC strain 43300. The ratio iso/anteiso varied between 0.119 (strain 531) and 0.418 (strain 3), whereas strain 530 showed the highest ratio of unsaturated versus saturated fatty acids. 

**Figure 1:**
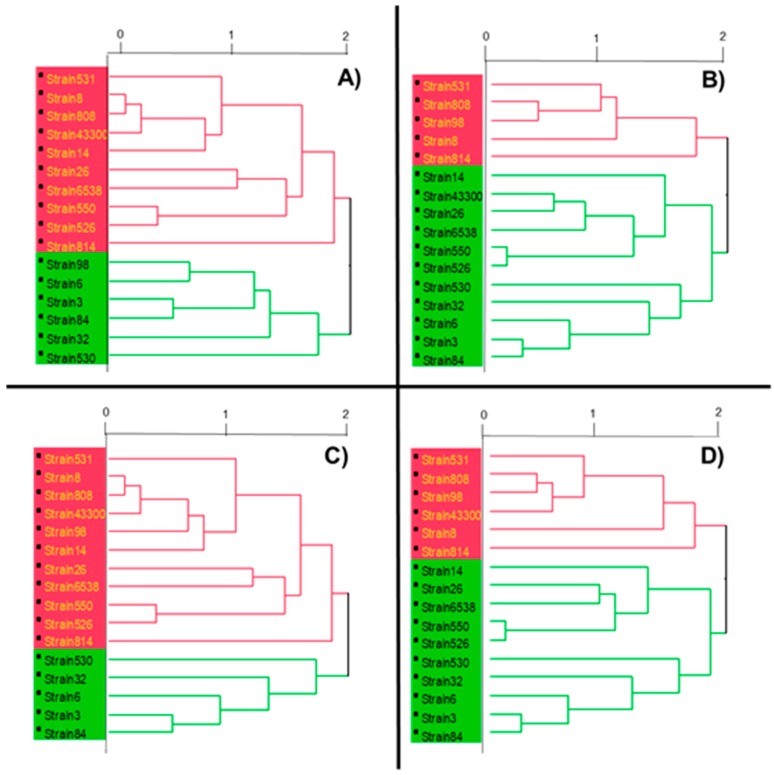
Dendrograms constructed using the Ward method for the cluster analysis of fatty acid from ATCC and clinical strains of *Staphylococcus aureus*: (**A**) fatty acid; (**B**) fatty acid and antibiotic susceptibility profile; (**C**) fatty acid and hydrophobicity index; (**D**) fatty acid, antibiotic susceptibility profile, and hydrophobicity index. The horizontal axis of the dendrogram represents the distance or dissimilarity between clusters. The vertical axis represents the objects and clusters. The horizontal axis is calculated as “even spacing”, so 2 represents the maximal distance, 1 the intermediate distance and 0 no distance.

The dendrogram reported in Figure 1A shows the distinction in two clusters (red and green) of the tested strains in relation to the amounts of bacterial lipids identified. 

### 2.2. Antibiotic Resistance

The classification of the strains into “susceptible standard dosing regimen” (S), “susceptible increased exposure” (I), and “resistant” (R) to the selected antibiotics according to Reference [15] is reported in Table 3. Results of negative controls indicated the complete absence of inhibition for all the strains (results not shown). All tested strains were susceptible to vancomycin, teicoplanin (with the exception of strain 6), and linezolid, whereas resistance was observed against benzyl penicillin. 

The minimum inhibitory concentration (MIC) and the minimum bactericidal concentration (MBC) values of vancomycin and teicoplanin are shown in Table 4. The detected MBC values were in the range between 0.31 and 1.25 μg/mL and 0.15 and 1.25 μg/mL for vancomycin and teicoplanin, respectively.

### 2.3. Cell-Surface Hydrophobicity

The bacteria tested showed a broad range of cell-surface hydrophobicity (expressed as hydrophobicity index %, Table 5). The lowest hydrophobicity indices (%) were obtained with the MRSA strains 526, 531, and 550 obtained from the pharynges. As for the lipid profile, strain 530 was significantly different from other strains isolated from pharynges, with higher hydrophobicity index. The two strains of *S. aureus* obtained from duodenal ulcers (strains 8, 14) showed a very similar hydrophobicity index, as well as strains 26 and 814 obtained from the pharynges. The highest hydrophobicity index was obtained with the ATCC strain 6538P, followed by the clinical strains isolated from the pharynges (3, 6, and 32).

### 2.4. Relation between Bacterial Lipid and Antibiotic Resistance

The dendrogram reported in Figure 1B shows the taxonomic relationships between bacterial lipid profiles and the antibiotic resistance across the tested strains. The bacteria closely related were classified into two groups (red and green); it is worth noting that the four strains obtained from a hip prostheses (strain 3, 6, 32, 84), as well the two ATCC strains, four (26, 550, 526, 530) out of seven strains from the pharynges, and one strain from ulcera (14) seemed closely related in terms of lipid profiles and antibiotic resistance, whereas all the other strains were grouped together. In particular, strains 3 and 84, both obtained from protheses, were similar in terms of lipid profile and antibiotic susceptibility (Figure 1A,B). Statistical analysis confirmed significant differences (*p* < 0.05) across the two groups on the relative abundance of the following lipids: acid 9-octadecenoic (C_18:1n-9_), acid 7-octadecenoic (C_18:1n-7_) and *iso*-nonadecanoic acid (*iso*-C_19:0_) (higher in the green group), eicosanoic acid (C_20:0_) (higher in the green group), henicosanoic acid (C_21:0_), and isohenicosanoic acid (*iso*-C_21:0_) (higher in the green group).

### 2.5. Relation between Bacterial Lipid and Cell-Surface Hydrophobicity

Figure 1C shows the dendrogram with the taxonomic relationships between bacterial lipid profiles and cell-surface hydrophobicity across the tested strains. Interestingly, the division into two groups did not vary with Figure 1C compared with Figure 1A, with the exception of strain 98, indicating no effect of cell-surface hydrophobicity on bacterial lipid profile. Statistical analysis also confirmed significant differences across the two groups as observed for antibiotic resistance.

### 2.6. Relation between Bacterial Lipid, Antibiotic Resistance and Cell-Surface Hydrophobicity

The dendrogram reported in Figure 1D shows the taxonomic relationships between bacterial lipid profiles, antibiotic resistance, and cell-surface hydrophobicity across the tested strains. The inclusion of the third parameter did not affect either the grouping or the statistical analysis compared with Figure 1B, with the exception of strain ATCC 43300.

## 3. Discussion

The present study reports the identification of lipid profiles of two ATCC strains (6538P and the methicillin-resistant 43300) and 14 clinical isolates of *S. aureus* and their implication on antibiotic resistance and cell-surface hydrophobicity. This was accomplished using a fast, comprehensive two-dimensional gas chromatography method in conjunction with mass spectrometry. 

It is well accepted that bacterial resistance to antibiotics has become an increasing threat, especially for MRSA strains [16]. This requires both the development of novel suitable drugs to overcome antibiotic resistance, including natural compounds, as well as a better understanding of the cellular response. Yadav et al. [6] have recently demonstrated that eugenol was effective against methicillin-resistant and methicillin-sensitive *Staphylococcus aureus* clinical strain biofilms. The effect of cranberry extracts has also been evaluated on the growth and biofilm production of *Escherichia coli* and *Staphylococcus* sp. [17].

We have recently shown that a white grape juice (*Vitis vinifera*) extract was effective against Gram-positive strains, including *S. aureus* ATCC 6538P and *S. aureus* ATCC 43300 [18]. The investigation of the changes in lipid distribution in *E. coli* strains in response to norfloxacin has detailed a lipid response resulting from the exposure of three closely-related strains to norfloxacin [12]. The functionality of lipid profiles in bacterial membranes has been widely reported, especially for Gram-negative bacteria. 

A recent study by Lopalco et al. [19] leads to the identification of unique cardiolipin and monolysocardiolipin species in *Acinetobacter baumannii* using combined matrix assisted laser desorption ionization-time of flight mass spectrometry (MALDI-TOF/MS) and thin-layer chromatography (TLC) analyses. A detailed knowledge on the glycerophospholipids present in the lipid extract of *A. baumannii,* including lipid classes, their proportion in membrane, and chain composition of lipids could help for the screening of possible mutants or specific membrane domains. The same authors had previously reported the presence of diphytanylglycerol analogues of phosphatidylinositol and phosphatidylglycerol in *Archea* bacteria [20]. 

Here, we demonstrated that bacterial lipid profiles varied both qualitatively and quantitatively across different strains of *S. aureus* and this reported variation affected both antibiotic resistance and cell-surface hydrophobicity. Based on the bacterial lipid profiles, we were able to identify common lipids, which were present and abundant across the strains, as well as characteristic lipids, which were not present and/or are less abundant across the strains. In particular, all clinical strains obtained from hip prostheses showed more resistance to quinolones compared to other antibiotics, which correlated with the presence of higher concentrations of C_18:1n-9_ and *iso*-C_21:0_. Two of these strains (6 and 32) also showed the highest cell-surface hydrophobicity index. On the other hand, most of the strains originated from the pharynges (526, 531, 550, 814) generally possessed low cell-surface hydrophobicity indexes. These strains were grouped together with the MRSA ATCC 43300 strain and had higher amounts of the characteristic lipids *anteiso*-pentadecanoic acid (*anteiso*-C_15:0_), *iso*-heptadecanoic acid (*iso*-C_17:0_), acid 7-octadecenoic (C_18:1n-7_), *iso*-nonadecanoic acid (*iso*-C_19:0_), and heneicosanoic acid (C_21:0_). Therefore, we can hypothesize a correlation between *anteiso*-C_15:0_, *iso*-C_17:0_, C_18:1n-7_, *iso*-C_19:0_, and C_21:0_ in bacterial membranes and methicillin resistance. 

The antibiotic resistance also seemed to be related to the source of infection the strains were isolated from; this could reflect their potential ability to create biofilm, whose eradication has proven rather difficult through common antibiotic treatments. The major adaptive response of the cells to an antimicrobial is to keep the fluidity of their membranes at a constant value, irrespective of the adverse environmental conditions. Such stabilization represents the main response of bacteria to membrane-active compounds or changes in the environmental conditions [21], thus preventing the loss of the physico-chemical properties of the lipid bilayer [22]. Mishra and Bayer [23] compared the lipid composition of daptomycin-susceptible and resistant MRSA; the results confirmed that daptomycin-resistant strains exhibited significantly reduced phosphatidylglycerol and carotenoid content. 

Variations of external conditions, such as temperature, pH, ethanol concentration, and osmolarity, as well as the presence of compounds affecting the microbial growth, with potential transition to the stationary phase, could result in the alteration of the fatty acid content controlling the membrane viscosity [8,24].

In particular, the increase of lipid unsaturation has been reported for several microbial cells as a consequence of the decrease of growth temperature; this effect seems related to the fatty acid synthesis mechanism utilized by the cell [25]. 

Fozo et al. [26] suggested that monounsaturated membrane fatty acids are necessary to maintain a pH across the membrane and an increase in fatty acid length within the cell membrane is associated to increase survival in acidic environments.

Di Pasqua et al. [27] have demonstrated that addition of antimicrobial compounds such as thymol, carvacrol, limonene, cinnaldeyde, and eugenol in the growing media of *Escherichia coli* strain O157:H7, *Salmonella enterica*, *Pseudomonas aeruginosa*, *Brochothrix thermosphacta*, and *Staphylococcus aureus* altered the fatty acid profiles of the strains. These findings could explain the changes in bacterial profiles shown in the present work in relation to antibiotic resistance. Addition of a sublethal concentration of the antimicrobial compounds resulted in an adaptation mechanism for the cells to maintain membrane structure and function [27]. 

It is well established that transport across the bacterial cell membranes is affected by their phase transition temperature [28]. The transport of branched-chain amino acids in *Pseudomonas aeruginosa* [29] requires phospholipids and depends on their acyl-chain length. 

## 4. Methods

### 4.1. Microbial Strains and Culture Conditions

The following strains were obtained from an in-house culture collection (University of Messina, Messina, Italy): *S. aureus* ATCC 6538P, *S. aureus* ATCC 43300, seven clinical strains of *S. aureus* obtained from the pharynges (strains 26, 526, 530, 531, 550, 808, 814), two clinical strains of *S. aureus* obtained from duodenal ulcers (strains 8, 14), four clinical strains of *S. aureus* obtained from hip prostheses (strains 3, 6, 32, 84), one clinical strain of *S. aureus* obtained from the conjunctiva (strain 98). 

Strains were cultured in Muller Hinton Broth (MHB, Oxoid, CM0405) at 37 °C (24 h) and cells washed three times in filtered phosphate-buffered saline (PBS) before lipid analysis.

### 4.2. Analysis of Bacterial Lipid Profiles 

Sample preparation of bacterial lipid profiles were performed as previously reported [14]. Briefly, bacterial lysis and transesterification of the fatty acids (FAs) were carried out with a simple one-step method. The methylated esters solution was then directly injected into the GC systems. The analyses were performed in triplicate.

The GC-MS and GC-FID runs were carried out on two parallel GC-QP2010 and GC2010 instruments (Shimadzu, Kyoto, Japan). The GC column used was a 30 m × 0.25 mm i.d. × 0.25 μm d_f_ Supelcowax-10 column (Sigma-Aldrich/Supelco, Bellefonte, PA, USA). Helium was used as the carrier gas at a constant linear velocity of 30.0 cm/s corresponding to an inlet pressure of 94.2 kPa for GC-MS and 97.4 kPa for GC-FID. Injection volume: 2 µL, split 10:1. The temperature program was the same in both analysis types: 50 °C to 280 °C at 3 °C/min, held for 5 min. 

The GC-MS ion source temperature was set at 200 °C; the interface temperature, 250 °C. The scan range was set to *m*/*z* 40–360, with a scanning rate of 1666 amu/s. The LIPIDS Mass Spectral Database (Shimadzu) was used for peak assignment, applying two filters, i.e., the spectrum similarity match over 85% and linear retention index (LRI) (related to a C_4_-C_24_ fatty acid methyl esters, FAMEs mixture) agreement in the ±10 range.

The FID temperature was set at 280 °C (sampling rate 40 ms) and gas flows were 40 mL/min for hydrogen and 400 mL/min for air.

The data handling was supported using GCMS solution ver. 2.6 and GC solution software (version 2.43, Shimadzu) for GC-MS and GC-FID analysis, respectively.

All the reagents, as well as the bacterial fatty acid methyl esters (BAMEs), were purchased from Millipore Sigma/Supelco (Bellefonte, PA, USA). 

### 4.3. Susceptibility Assays

The agar diffusion test (Kirby–Bauer antibiotic test) was used to investigate the extent to which the tested bacteria were affected by the following antibiotics: oxacillin (1 μg), clarithromycin (15 μg), levofloxacin (5 μg), cefoxitin (30 μg), vancomycin (30 μg), teicoplanin (30 μg), tetracycline (30 μg), erythromycin (15 μg), gentamicin (10 μg), clindamycin (2 μg), trimethoprim-sulfamethoxazole (25 μg), rifampicin (30 μg), linezolid (10 μg), benzylpenicillin (1 UI). The zone of inhibition (mm) was measured and recorded according to The European Committee on Antimicrobial Susceptibility Testing (EUCAST) [15]. The assay was performed in triplicate. The minimum inhibitory concentrations (MIC) and the minimum bactericidal concentrations (MBC) of vancomycin and teicoplanin against all tested strains were determined using a broth microdilution according to the Clinical and Laboratory Standards Institute [30]. MIC values were defined as the lowest extract concentrations showing no bacterial growth after the incubation time. MBCs were determined by seeding an inoculum (20 μL) derived from clear MIC wells onto Mueller–Hinton agar (MHA, Oxoid) plates. The MBC was defined as the lowest extract concentration, which killed 99.9% after 24 h incubation at 37 °C. All assays were performed in triplicate.

### 4.4. Cell-Surface Hydrophobicity 

The cell-surface hydrophobicity of *S. aureus* strains was measured as described by Martin et al. [31]. Bacterial cells, grown overnight in MHB medium at 37 °C, were centrifuged, washed three times, and suspended in sterile saline (0.85%) until their optical density was 0.3 at 600 nm. The cell suspension (6 mL) was thereafter placed in tubes, where toluene (1 mL) was added. The tubes were uniformly mixed for 2 min and allowed to equilibrate at room temperature for 10 min. After separation of the toluene phase from the aqueous phase, the optical density (OD) of the aqueous phase was determined spectrophotometrically at 600 nm. The hydrophobicity index (HPBI) was calculated as follows:

(1) $$\frac{\mathrm{OD}\;\left(\mathrm{initial}\right)-\mathrm{OD}\;\left(\mathrm{final}\right)}{\mathrm{OD}\;\left(\mathrm{initial}\right)}\;\times \;100$$

### 4.5. Data Analysis

Analytical data were processed using the statistical JMP7 for SAS software (version 7, SAS Institute Inc., Cary, NC, USA). All analyses were performed in triplicate and repeated at least three times (*n* = 3). Data were recorded as mean ± standard deviation (SD). Cluster analysis was performed, using all the variables studied, through multivariate analysis by Ward’s method.

### 4.6. Statistical Analysis

A cluster analysis was performed to identify groups of bacteria for each feature (only lipids, lipids + antibiotic susceptibility profile, lipids + hydrophobicity, lipids + antibiotic susceptibility profile + hydrophobicity), such that two groups have been identified for each category. Based on the cluster analysis results, a comparison between groups was obtained. Examined variables were not normally distributed, such as verified via the Kolmogorov Smirnov test; consequently, the non-parametric approach was used. The Mann–Whitney test was applied in order to identify the variables either discriminating or common to the examined groups.

Statistical analyses were performed using SPSS 17.0 for Windows package (IBM, Armonk, NY, USA). For statistical significance, *p* < 0.05 (two-sided) was considered significant.

## 5. Conclusions

The results of the present study indicate a potential correlation between the lipid profile of Gram-positive strains of *S. aureus*, site of infection, antibiotic resistance, and cell-surface hydrophobicity. This was achieved using a GC-MS method to identify fatty acids in bacterial lipid extracts. Further studies will be aimed at identifying the molecular mechanisms involved in the correlation between lipid composition and antibiotic resistance.

## Figures and Tables

**Table 1:** Bacterial fatty acid methyl esters (BAMEs) quantified (%) in bacterial strains under study by Gas Chromatography-Flame Ionization Detector (GC-FID) analysis. n.d.= not detected; tr = traces.

BAMEs	Bacterial Strain							
	531	26	808	43300	8	814	530	14
Me. C_6:0_	n.d.	n.d.	n.d.	n.d.	n.d.	n.d.	n.d.	0.11
Me. C_10:0_	n.d.	n.d.	n.d.	n.d.	n.d.	n.d.	n.d.	n.d.
Me. C_11:0_	n.d.	n.d.	n.d.	n.d.	n.d.	n.d.	n.d.	n.d.
Me. C_12:0_	n.d.	n.d.	n.d.	0.05	n.d.	n.d.	0.49	n.d.
Me. C_13:0_ iso	0.05	0.21	n.d.	0.05	n.d.	0.10	0.29	n.d.
Me. C_13:0_	n.d.	n.d.	n.d.	n.d.	n.d.	n.d.	n.d.	n.d.
Me. C_13:0_ anteiso	0.16	0.03	n.d.	0.06	n.d.	n.d.	0.10	n.d.
Me. C_14:0_ iso	0.86	0.65	0.97	0.49	1.32	1.71	0.78	0.84
Me. C_14:0_	0.15	0.08	0.21	0.19	0.33	0.39	1.74	0.65
Me. C_15:0_ iso	3.66	4.83	6.81	5.84	9.41	10.23	4.97	5.69
Me. C_15:0_ anteiso	58.98	50.98	52.97	52.30	56.04	49.95	33.32	48.42
Me. C_15:0_	0.03	0.05	n.d.	tr	n.d.	n.d.	0.10	n.d.
Me. C_16:0_ anteiso	n.d.	2.08	n.d.	n.d.	n.d.	n.d.	n.d.	n.d.
Me. C_16:0_ iso	2.18	n.d.	2.20	1.23	2.00	2.70	2.82	1.98
Me. C_16:0_	1.44	0.95	1.93	1.63	2.39	2.35	1.70	3.73
Me. C_17:0_ iso	2.33	4.24	4.13	3.80	4.13	4.40	4.20	3.65
Me. C_17:0_ anteiso	22.98	22.64	18.73	21.18	14.16	13.76	17.25	21.04
Me. C_17:0_	0.08	0.16	n.d.	0.08	0.10	0.15	1.60	n.d.
Me. C_18:0_ iso	0.49	1.13	0.80	0.50	0.63	0.83	1.39	0.88
Me. C_18:0_	2.68	3.14	4.52	6.10	4.50	4.41	6.15	6.17
Me. C_18:1n6_	n.d.	n.d.	n.d.	n.d.	n.d.	0.97	n.d.	n.d.
Me. C_18:1n9_	0.18	n.d.	1.93	n.d.	n.d.	n.d.	0.78	n.d.
Me. C_18:1n7_	n.d.	n.d.	n.d.	n.d.	n.d.	0.06	0.12	n.d.
Me. C_18:1n3_	n.d.	n.d.	n.d.	n.d.	n.d.	n.d.	n.d.	n.d.
Me. C_18:1n11_	0.05	0.35	n.d.	n.d.	n.d.	n.d.	n.d.	n.d.
Me. C_19:0_ iso	0.50	1.51	1.08	1.08	0.94	n.d.	1.90	0.90
Me. C_18:2n6_	n.d.	n.d.	n.d.	n.d.	n.d.	0.83	n.d.	n.d.
Me. C_18:2n9_	0.23	n.d.	n.d.	n.d.	n.d.	n.d.	n.d.	n.d.
Me. C_18:3n3_	n.d.	n.d.	n.d.	n.d.	n.d.	n.d.	12.04	n.d.
Me. C_19:0_ anteiso	2.31	4.64	2.63	3.51	1.99	1.53	4.24	3.55
Me. C_19:0_ iso	n.d.	n.d.	n.d.	n.d.	n.d.	0.93	0,36	n.d.
Me. C_19:0_	0.05	0.20	n.d.	0.14	0.27	0.36	n.d.	0.16
Me. C_19:3n3_	n.d.	n.d.	n.d.	n.d.	n.d.	2.70	n.d.	n.d.
Me. C_20:0_	0.61	1.78	1.08	1.61	1.79	1.53	3.10	1.90
Me. C_20:0_ iso	n.d.	0.36	n.d.	0.09	n.d.	0.12	0.31	0.31
Me. C_20:1n7_	n.d.	n.d.	n.d.	n.d.	n.d.	n.d.	n.d.	n.d.
Me. C_20:1n9_	n.d.	n.d.	n.d.	0.06	n.d.	n.d.	n.d.	n.d.
Me. C_21:0_	n.d.	n.d.	n.d.	n.d.	n.d.	n.d.	n.d.	n.d.
Me. C_21:0_ iso	n.d.	n.d.	n.d.	n.d.	n.d.	n.d.	0.26	n.d.
Me. C_22:0_	n.d.	n.d.	n.d.	n.d.	n.d.	n.d.	n.d.	n.d.
Me. C_22:1n9_	n.d.	n.d.	n.d.	n.d.	n.d.	n.d.	n.d.	n.d.
Me. C_22:1n11_	n.d.	n.d.	n.d.	n.d.	n.d.	n.d.	n.d.	n.d.
Me. C_24:0_	n.d.	n.d.	n.d.	n.d.	n.d.	n.d.	n.d.	n.d.
MEs	Bacterial Strains							
	550	32	6538P	526	6	3	98	84
Me. C_6:0_	n.d.	n.d.	n.d.	n.d.	n.d.	n.d.	n.d.	n.d.
Me. C_10:0_	n.d.	n.d.	n.d.	n.d.	0.09	n.d.	0.11	n.d.
Me. C_11:0_	n.d.	n.d.	n.d.	n.d.	n.d.	n.d.	n.d.	n.d.
Me. C_12:0_	0.13	0.03	0.27	0.03	0.02	0.06	0.09	0.07
Me. C_13:0_ iso	0.17	0.09	0.14	0.29	0.10	0.22	0.12	0.13
Me. C_13:0_	n.d.	n.d.	n.d.	n.d.	n.d.	n.d.	n.d.	n.d.
Me. C_13:0_ anteiso	0.50	0.06	0.11	0.06	0.06	0.06	0.07	0,06
Me. C_14:0_ iso	1.24	0.64	0.75	1.16	0.56	1.22	0.79	1.16
Me. C_14:0_	0.36	0.16	0.43	0.26	0.18	0.18	0.29	0.22
Me. C_15:0_ iso	6.53	5.84	6.00	5.79	9.38	9.11	n.d.	9.24
Me. C_15:0_ anteiso	44.53	44.42	45.04	47.80	41.91	38.35	47.93	43.99
Me. C_15:0_	0.09	0.03	0.03	0.14	0.02	n.d.	0.01	0.03
Me. C_16:0_ anteiso	n.d.	n.d.	1.81	n.d.	n.d.	n.d.	n.d.	n.d.
Me. C_16:0_ iso	2.47	1.20	n.d.	2.49	1.57	2.70	1.61	2.49
Me. C_16:0_	3.41	1.60	4.38	4.10	1.91	1.98	2.20	2.13
Me. C_17:0_ iso	4.44	4.54	3.70	3.93	6.87	7.35	5.27	6.01
Me. C_17:0_ anteiso	18.08	20.76	19.84	18.49	19.20	17.35	20.95	18.06
Me. C_17:0_	n.d.	0.16	0.22	0.11	0.08	0.09	0.11	2.03
Me. C_18:0_ iso	1.29	0.74	0.78	1.12	0.80	1.46	0.64	0.92
Me. C_18:0_	7.82	7.53	7.50	7.32	6.53	7.13	5.22	6.45
Me. C_18:1n6_	n.d.	n.d.	n.d.	n.d.	n.d.	n.d.	n.d.	n.d.
Me. C_18:1n9_	n.d.	0.19	n.d.	n.d.	0.57	1.29	0.60	0.92
Me. C_18:1n7_	n.d.	0.04	n.d.	n.d.	0.06	0.13	0.09	0.07
Me. C_18:1n3_	n.d.	0.01	n.d.	n.d.	n.d.	n.d.	n.d.	n.d.
Me. C_18:1n11_	n.d.	n.d.	n.d.	n.d.	n.d.	n.d.	n.d.	n.d.
Me. C_19:0_ iso	1.46	1.74	1.19	n.d.	2.13	2.16	1.11	1.03
Me. C_18:2n6_	n.d.	n.d.	n.d.	n.d.	n.d.	n.d.	n.d.	n.d.
Me. C_18:2n9_	n.d.	n.d.	n.d.	n.d.	n.d.	n.d.	n.d.	n.d.
Me. C_18:3n3_	n.d.	tr	n.d.	n.d.	n.d.	n.d.	n.d.	n.d.
Me. C_19:0_ anteiso	3.92	5.22	4.19	3.67	3.72	3.71	3.20	2.36
Me. C_19:0_ iso	0.22	n.d.	n.d.	1.11	n.d.	n.d.	n.d.	n.d.
Me. C_19:0_	n.d.	0.34	0.13	0.09	0.16	0.36	0.13	0.22
Me. C_19:3n3_	n.d.	n.d.	n.d.	n.d.	n.d.	n.d.	n.d.	n.d.
Me. C_20:0_	2.89	4.01	2.15	1.71	3.28	4.15	1.84	1.90
Me. C_20:0_ iso	0.46	0.21	0.26	0.33	0.22	0.50	0.20	0.18
Me. C_20:1n7_	n.d.	0.03	n.d.	n.d.	n.d.	n.d.	n.d.	n.d.
Me. C_20:1n9_	n.d.	0.07	n.d.	n.d.	n.d.	n.d.	n.d.	n.d.
Me. C_21:0_	n.d.	0.25	n.d.	n.d.	0.16	0.31	n.d.	0.18
Me. C_21:0_ iso	n.d.	0.10	1.08	n.d.	0.17	0.14	n.d.	0.18
Me. C_22:0_	n.d.	n.d.	n.d.	n.d.	0.26	n.d.	n.d.	n.d.
Me. C_22:1n9_	n.d.	n.d.	n.d.	n.d.	n.d.	n.d.	n.d.	n.d.
Me. C_22:1n11_	n.d.	n.d.	n.d.	n.d.	n.d.	n.d.	n.d.	n.d.
Me. C_24:0_	n.d.	n.d.	n.d.	n.d.	n.d.	n.d.	n.d.	n.d.

**Table 2:** Features of lipid profiling identified in the selected bacterial strains.

Bacterial Strain	Branched Chain/Straight Chain	Iso/Anteiso	UFAs/SFAs	FA Chain Length Ranges
531	17.179	0.119	0.005	C13-C20
26	13.932	0.161	0.004	C13-C20
581	9.777	0.162	0.011	C11-C24
808	9.333	0.215	0.020	C14-C20
ATCC 43300	9.125	0.170	0.001	C12-C20
8	9.663	0.255	0.000	C14-C20
814	6.281	0.322	0.048	C13-C20
530	2.595	0.314	0.150	C12-C21
14	6.854	0.195	0.000	C6-C20
550	5.803	0.273	0.000	C12-C20
32	5.919	0.214	0.003	C12-C21
ATCC 6538P	5.615	0.196	0.000	C12-C21
526	6.273	0.232	0.000	C12-C20
6	6.512	0.336	0.006	C10-C22
3	5.376	0.418	0.014	C12-C21
98	7.667	0.135	0.008	C10-C20

Iso/Anteiso represents the ratio iso/anteiso (branched chain). UFA/sFAs represents the ratio unsaturated fatty acids (UFAs)/saturated fatty acids (sFAs). FA: fatty acid.

**Table 3:** Strains classification into “susceptible standard dosing regimen” (S), “susceptible increased exposure” (I), and “resistant” (R) to the selected antibiotics. Values represent the mean of three determinations ± SD. R indicates resistance.

Strain	Oxacillin	Cefoxitin	Clarithromycin	Vancomycin	Levofloxacin	Teicoplanin	Tetracycline	Erythromycin	Gentamicin	Clindamycin	Trimethoprim-Sulfamethoxazole	Rifampicin	Linezolid	Benzylpenicillin
8	S	S	I	S	S	S	S	S	S	S	R	I	S	R
98	S	R	I	S	S	S	I	S	R	R	R	R	S	R
6	R	S	I	S	S	R	S	R	S	I	S	S	S	R
ATCC 43300	R	R	R	S	S	S	S	R	S	R	S	S	S	R
84	S	S	R	S	S	S	S	R	S	I	S	S	S	R
530	R	R	I	S	S	S	S	S	S	I	R	S	S	R
32	S	S	R	S	S	S	S	R	S	S	S	S	S	R
808	S	S	I	S	S	S	S	S	S	S	S	S	S	R
ATCC 6538P	S	S	I	S	S	S	S	S	S	S	S	S	S	R
3	S	S	I	S	S	S	S	S	S	I	S	S	S	R
814	R	S	I	S	S	S	S	I	S	I	S	S	S	R
14	S	S	I	S	S	S	S	R	S	I	S	S	S	R
26	S	S	I	S	S	S	R	S	S	S	R	S	S	R
526	S	S	I	S	S	S	S	S	R	I	S	S	S	R
550	R	S	I	S	S	S	S	S	S	S	S	S	S	R
531	S	S	I	S	S	S	S	S	R	R	S	S	S	R

**Table 4:** MICs (μg mL^−1^) and MBC (μg mL^−1^) of vancomycin and teicoplanin against ATCC and clinical strains of *S. aureus*.

Strain	Vancomycin		Teicoplanin	
	MIC	MBC	MIC	MBC
8	0.62–1.25	1.25	0.31	0.31
98	0.31	0.31	0.31	0.31
6	0.31	0.62	1.25	1.25
ATCC 43300	0.31	0.62	0.31	0.31
84	0.62	0.62	0.62	1.25
530	0.31–0.62	0.62	0.31–0.62	0.62
32	0.62	0.62	0.31	0.31
808	0.31	0.31	0.15	0.15
ATCC 6538P	0.31	0.62	0.15	0.15
3	0.31	0.62	0.15–0.31	0.31
814	0.62	0.62	0.62	1.25
14	0.31	0.31	0.15–0.31	0.31
26	0.62	0.62	0.31	0.62
526	0.31	0.62	0.31	0.62
550	0.31	0.62	0.31	1.25
531	0.31	0.31	0.078–0.15	0.62

**Table 5:** Cell-surface hydrophobicity (expressed as % hydrophobicity index) of ATCC and clinical strains of *S. aureus*. Values represent the mean of three determinations.

Strain	Hydrophobicity Index (%)
8	23.89 ± 1.7
98	62.76 ± 2.5
6	63.29 ± 1.9
ATCC 43300	57.23 ± 2.4
84	47.66 ± 1.5
530	50.50 ± 2.8
32	65.13 ± 3.4
808	67.05 ± 4.2
ATCC 6538P	80.23 ± 3.6
3	64.11 ± 2.4
814	32.06 ± 2.6
14	35.00 ± 2.8
26	31.37 ± 3.4
526	8.68 ± 2.5
550	10.47 ± 1.2
531	3.33 ± 0.8

## Abbreviations

*S. aureus*	*Staphylococcus aureus*
*anteiso*-C_15:0_	*anteiso*-pentadecanoic acid
*anteiso*-C_17:0_	*anteiso*-heptadecanoic acid
*iso*-C_15:0_	*iso*-pentadecanoic acid
*iso*-C_13:0_	*Iso*-tridecanoic acid
*anteiso*-C_13:0_	*anteiso*-tridecanoic acid
*iso*-C_16:0_	*iso*-hexadecanoic acid
*anteiso*-C_16:0_	*anteiso*-hexadecanoic acid
C_18:0_	octadecanoic acid
MRSA	methicillin-resistant *S. aureus*
GC×GC-MS	gas chromatography in combination with mass spectrometry
FA	fatty acid
BAMEs	bacterial fatty acid methyl esters
